# Supplementary material for: Diagnostic Accuracy of Non-Contrast-Enhanced Time-Resolved MR Angiography to Assess Angioarchitectural Classification Features of Brain Arteriovenous Malformations
Source: Diagnostics (Basel). 2024 Jul 31;14(15):1656. doi: 10.3390/diagnostics14151656 (PMC11311491; doi:10.3390/diagnostics14151656)
Supplement: Supplementary file 1 [file diagnostics-14-01656-s001.zip › diagnostics-3083398-supplementary.pdf]

Supplementary Materials

	Scores					
Criteria	Spetzler-Martin		AVMES		Buffalo	
Nidus size	< 3 cm	1	< 3 cm	1		
	3-6 cm	2	3-6 cm	2		
	> 6 cm	3	> 6 cm	3		
Eloquence	Noneloquent	0	Noneloquent	0	Noneloquent	0
	Eloquent	1	Eloquent	1	Eloquent	1
Venous drainage	Superficial only	0	≤ 3 draining veins	1		
	Deep	1	4-6 draining veins	2		
			>6 draining veins	3		
Arterial feeders			≤ 3	1	1 or 2	1
			4-6	2	3 or 4	2
			> 6	3	5 or more	3
					Most > 1 mm	0
					Most ≤ 1 mm	1
Total score range	1-5		3-10		1-5	

Supplementary Table S1. Spetzler-Martin, AVM Embocure Score and Buffalo grading scales

Acronym	Characteristics		Points
R2	Race	Nonwhite	0
		White	2
eD	Deep location	Nonexclusive	0
		Exclusive	1
A	AVM size	< 3 cm	0
		≥ 3 cm	1
V	Venous drainage	Exclusive deep	0
		Other	1
M	(Mono)arterial feeding	Unique	0
		> Feeding artery	1

Supplementary Table S2. R2eDAVM grading scale.
